# Gastrointestinal Kohlmeier–Degos disease: a narrative review

**DOI:** 10.1186/s13023-022-02322-9

**Published:** 2022-04-20

**Authors:** Samantha S. Sattler, Cynthia M. Magro, Lee Shapiro, Jamie F. Merves, Rebecca Levy, Jesse Veenstra, Puraj Patel

**Affiliations:** 1 Albany Medical College, Albany, NY USA; 2 Department of Pathology and Laboratory Medicine, Weill Cornell Medicine, New York, NY USA; 3 Division of Rheumatology, Albany Medical College, 6 Medical Park Drive, Malta, NY 12020 USA; 4 Division of Gastroenterology, Hepatology and Nutrition, Children’s Hospital of Philadelphia, Philadelphia, PA USA; 5 Division of Dermatology, Department of Pediatrics, The Hospital for Sick Children, Toronto, ON Canada; 6 Department of Dermatology, Henry Ford Health System, Detroit, MI USA; 7 Department of Surgery, Henry Ford Health System, Detroit, MI USA

**Keywords:** Kohlmeier–Degos disease, Degos disease, Gastrointestinal, Perforation, Laparoscopy, C5b-9, Interferon, Eculizumab, Treprostinil

## Abstract

**Introduction:**

Kohlmeier-Degos (K-D) disease is a rare obliterative vasculopathy that can present as a benign cutaneous form or with potentially malignant systemic involvement. The gastrointestinal tract is most frequently involved in systemic disease and mortality is often related to bowel perforations. Herein, we provide information to providers and patients regarding gastrointestinal K-D symptomology, pathology, treatment, and diagnosis, with a focus on the importance of timely diagnostic laparoscopy. We present three new cases of gastrointestinal K-D to highlight varying disease presentations and outcomes.

**Body:**

Based on reviewed reports, perforation is preceded by at least one gastrointestinal symptom: abdominal pain/cramping, anorexia/weight loss, vomiting, diarrhea, nausea, gastrointestinal bleeding, obstipation, constipation, and abdominal fullness. Perforation most commonly occurs in the small intestine and often results in sepsis and death. Although underutilized, laparoscopy is the most sensitive and specific diagnostic technique, demonstrating serosal porcelain plaques similar to those on the skin and characteristic for K–D. The combination of eculizumab and treprostinil is presently the most effective treatment option for gastrointestinal K–D. The pathology of gastrointestinal K-D is characterized by an obliterative intimal arteriopathy eventuating in occlusive acellular deposits of mucin and collagen along with an extravascular pauci-cellular sclerosing process resembling scleroderma confined to the subserosal fat. C5b-9 and interferon-alpha are both expressed in all caliber of vessels in the affected intestine. While C5b-9 blockade does not prevent the intimal expansion, enhanced type I interferon signaling is likely a key determinant to intimal expansion by, causing an influx of monocytes which transdifferentiate into procollagen-producing myofibroblast-like cells.

**Conclusion:**

Prompt laparoscopic evaluation is necessary in any K–D patient with an abdominal symptom to facilitate diagnosis and treatment initiation, as well as to hopefully decrease mortality. Those with gastrointestinal K–D should start on eculizumab as soon as possible, as onset of action is immediate.

**Supplementary Information:**

The online version contains supplementary material available at 10.1186/s13023-022-02322-9.

## Background

**Fig. 1:**
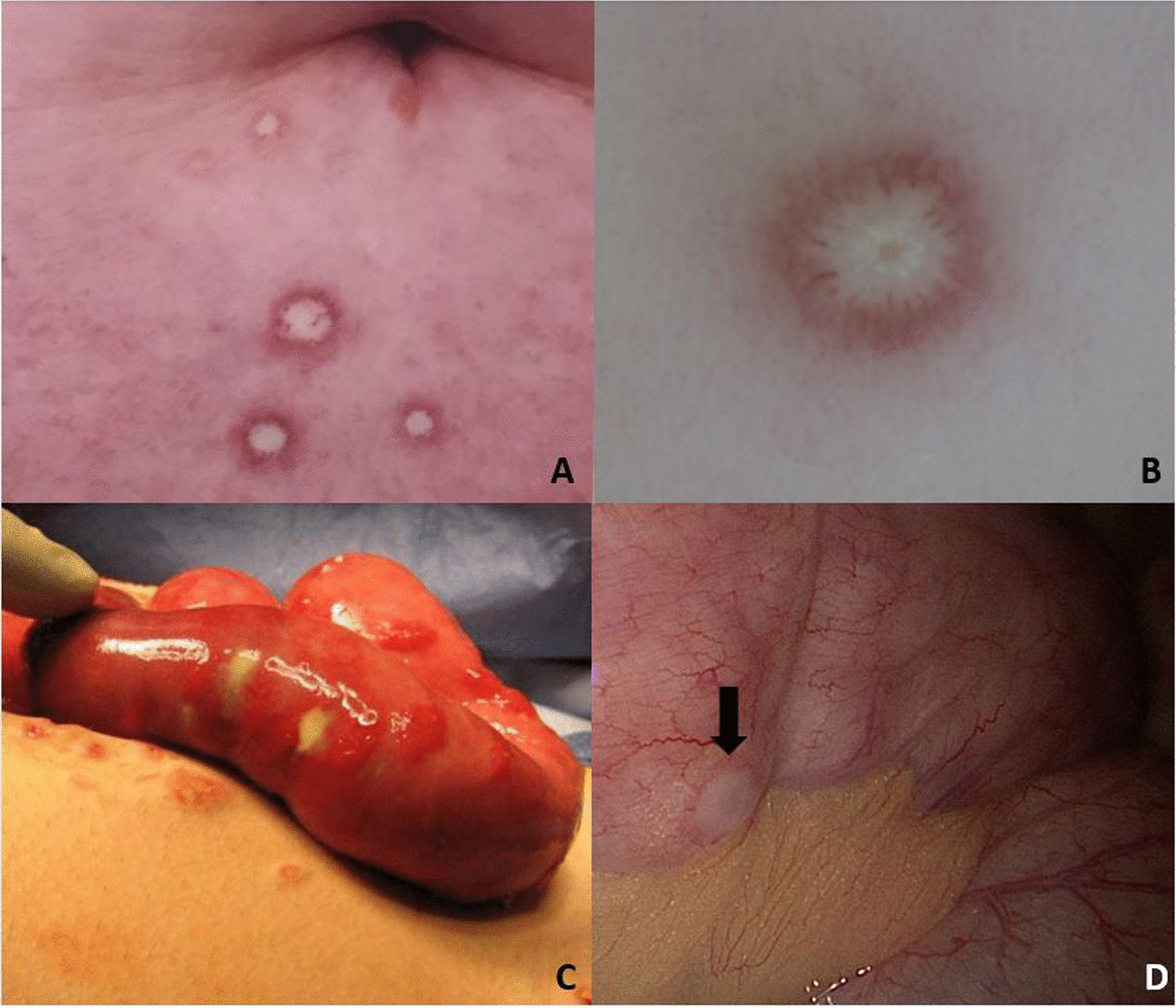
**A** Mature skin lesions. **B** Magnified skin lesions demonstrating an atrophic, avascular center with an erythematous border of telangiectasia. **C** Laparotomy showing the bowel serosa studded with porcelain-white lesions that resemble those on the skin (Copyright (2020) ACR). **D** Laparoscopy offers a less-invasive means of identifying these highly-specific serosal bowel lesions (arrow)

Kohlmeier–Degos (K–D) disease is a rare obliterative vasculopathy that can present with benign cutaneous lesions or as a potentially malignant systemic process (“malignant atrophic papulosis”). In cutaneous K–D, mature lesions demonstrate an atrophic and avascular “porcelain-white” center with an erythematous border, most often on the trunk and upper extremities (Fig. 1A) [1]. These lesions are frequently few in number and asymptomatic. They often spare the face and hands and thus, may go unnoticed. In the systemic form, visceral involvement may occur before cutaneous lesions have been recognized. Most patients with systemic K–D succumb to the disease within a few years [2]. Gastrointestinal, central nervous system, and pleural and pericardial manifestations have been noted. The gastrointestinal tract is most frequently involved [2], and mortality is most often related to bowel perforations [1]. Early recognition of this potentially life-threatening entity is essential to improve outcomes since successful interventions have been reported [3]. Sixty-five previously published cases of gastrointestinal K-D are herein reviewed, and three new cases are reported. Additionally, we focus on laparoscopic imaging as an effective diagnostic procedure and provide an overview of pathology and treatment.

## Methods

A literature review of gastrointestinal K–D was conducted. Additional file 1 demonstrates search methods. Articles included were those in which patients were diagnosed with K–D and reported at least one gastrointestinal symptom. In total, 58 publications were included [2–59].

## Results

Across the 58 included articles, 65 patient cases were described. Of the 63 cases in which gender was reported, 46% (n = 29) were females and 54% (n = 34) were males. Of the 62 cases in which age was reported, two occurred in infants and six in those younger than age 18 years; of the 54 cases in patients 18 or older, the median age of presentation was 42 years. Most reports did not document patient race.

In these 65 patient cases, K–D skin lesions preceded gastrointestinal manifestations, with numbers ranging from several to many hundred and patient awareness of lesions ranging from only a few days to almost 30 years [25]. The median time for onset of any systemic symptoms after the appearance of cutaneous lesions was one year [1].

K–D diagnoses were established via biopsy of distinctive cutaneous lesions in 54% (n = 35) of cases. Biopsies revealed wedge-shaped ischemia and overlying hyperkeratosis or lymphocytic inflammation around dermal venules and arterioles [60]. Two cases were diagnosed postmortem.

Various gastrointestinal techniques were employed in evaluation. Endoscopy most often showed ulcerations though patchy erythematous change and inflammation were also seen. Colonoscopy infrequently demonstrated ulceration and strictures [8]. Laparotomy was commonly performed and revealed ischemia, shallow ulcers, edema, purulent collections, and serosal yellow plaques. Laparoscopy offers a less-invasive means of identifying these highly specific lesions [4]. While the intestinal serosa is classically involved, non-intestinal serosal lesions may also occur, as has been seen on the liver capsule and peritoneum [22].

Of all 65 patient cases, 49% (n = 32) reported gastrointestinal perforation. Of these, only five had survived by the time of case publication [4, 13, 26, 46, 54]. Fifty percent of patients with perforation (n = 16) suffered from more than one concurrent perforation at the time of initial visualization. Across these 16 patients, there were a total of 42 perforations with distinct locations specified. Of all perforations in the cohort (n = 58), 74% (n = 43) occurred in the small intestine. Of those specified, four were duodenal, 16 jejunal, 17 ileal, 19% (n = 11) colonic (of those specified, two cecal, three in the ascending colon, one in the splenic flexure, and three sigmoidal), 5% (n = 3) gastric, and 1% esophageal (n = 1). In six patients, the location of perforation was not specified.

Each reported case of perforation was preceded by at least one gastrointestinal symptom, most frequently abdominal pain/cramping, the next most frequent being anorexia/weight loss and vomiting. Less common symptoms included diarrhea, nausea, and gastrointestinal bleeding, with infrequent reports of obstipation, constipation, and abdominal fullness. Perforation was not reported in a previously asymptomatic patient. The duration of time from abdominal symptom onset to perforation was variable and unpredictable. Most perforations were identified through emergency laparotomy or were recognized at autopsy. Patients and their physicians may have been unaware of the peril that gastrointestinal symptoms represent in the setting of K-D, may not have recognized existing skin lesions as K–D, or may not have known how to establish the diagnosis before perforation occurred.

Fifty-seven percent (n = 37) of gastrointestinal K–D occurred with other systemic manifestations. Of these, neurologic involvement was most common (n = 23, 40%). Synchronous neurologic and gastrointestinal symptoms (n = 10 of 23 gastrointestinal-neurologic K–D cases, 43%) or gastrointestinal manifestations preceding neurologic symptoms (n = 9, 39%) were common patterns. Infrequently, neurologic symptoms developed before gastrointestinal (n = 4, 17%). There was one case dominated by clinical gastrointestinal symptoms with neurologic disease found on autopsy [56] and one with primarily clinical neurologic symptoms and gastrointestinal disease found on autopsy [49].

Other systemic manifestations seen with gastrointestinal K–D included pleural (n = 16, 25%), pericardial (n = 10, 15%), renal (n = 8, 12%), and ophthalmic (n = 5, 8%). Of the five patients who survived following perforation, three had extra-abdominal systemic manifestations, with pleural and pericardial involvement [26] and neurologic involvement [38, 46].

K–D may coexist with dermatomyositis [4, 20, 34, 52], systemic lupus erythermatosus, antiphospholipid antibody syndrome, and systemic sclerosis.

We present two cases in which diagnosis by laparoscopy enabled successful intervention. In the third case, perforation occurred before this procedure was accomplished and the outcome was fatal.

## CASE 1

A 15-year-old male presented with lesions beginning 1.5 years prior. They began as asymptomatic papules that progressed to atrophic porcelain lesions with bright-pink erythematous rims. The lesions spread from his abdomen to his legs, increasing to over 100 in number.

**Fig. 2:**
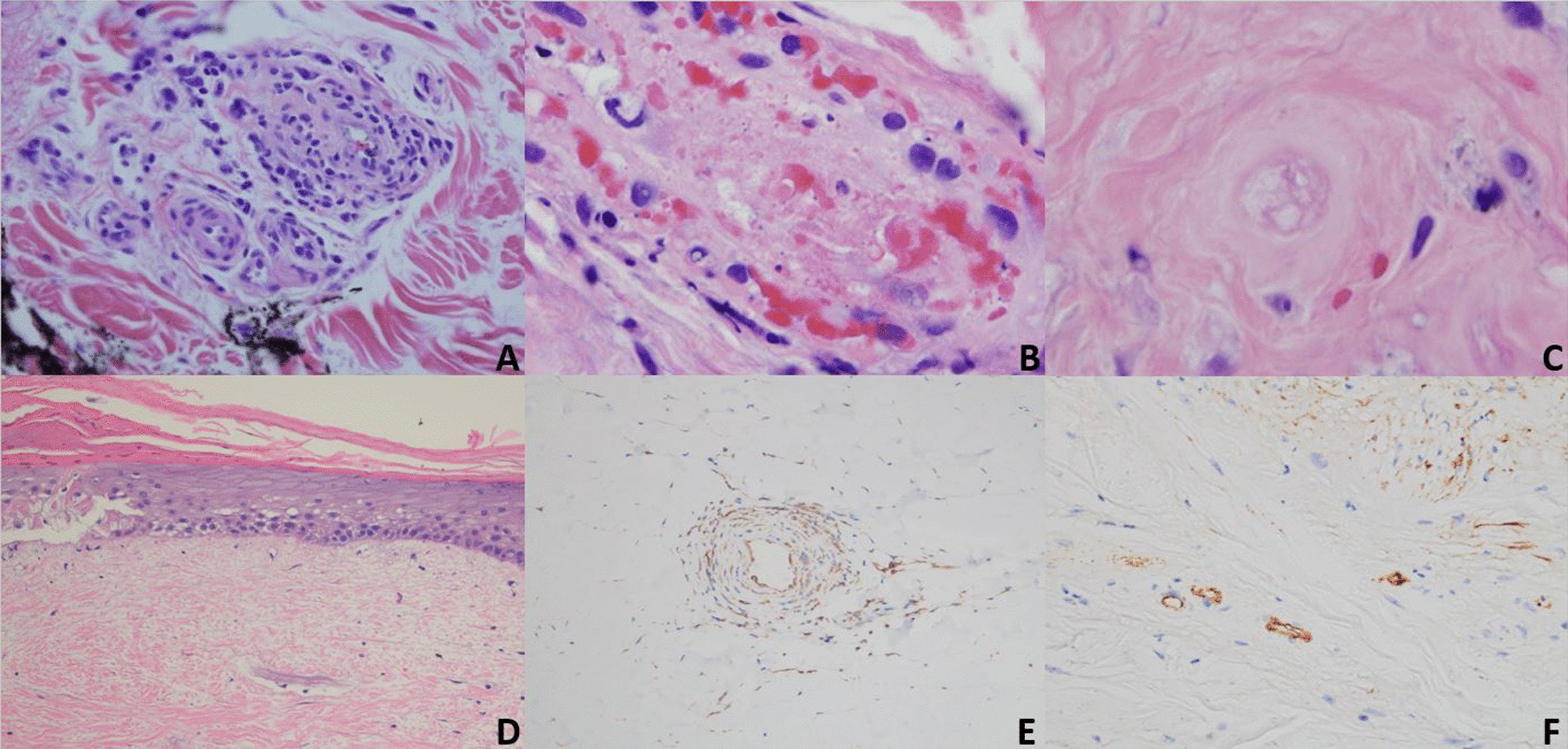
Chronologic pathology of cutaneous K-D as occurs in the microvasculature of the dermis: **A** Early inflammatory phase characterized by a vasocentric lymphocytic infiltrate permeative of the vessel wall with variable fibrin deposition and red cell extravasation consistent with a lymphocytic vasculitis (H&E, ×40). **B** Due to the targeted endothelial cell injury there is endothelial cell necrosis, subsequent denudement followed by vascular thrombosis with some inflammatory residuum eventuating into a relatively pauci-inflammatory thrombogenic vasculopathy (H&E, ×40). **C** Repetitive episodes of endothelial cell injury cause basement membrane zone thickening while the lining endothelium has a mummified anucleated appearance (H&E, ×40). **D** Eventually the vessels disappear and there is ensuing ischemic driven fibrosis corresponding clinically to the depressed porcelain white center (H&E, ×4). **E** Enhanced interferon expression as demonstrated by the extent of myxovirus A (MXA) deposition within the vessel wall, amidst inflammatory cells and endothelium (MXA, ×40). **F** There are extensive microvascular deposits of C5b-9 (C5b-9, ×10)

Biopsies showed morphology typical of K-D disease characterized by an attenuated epidermis and subepidermal wedge-shaped fibrosis with vascular drop-out (Fig. 2D). There were also microvessels outside the zone of hypovascular fibroplasia exhibiting an active lymphocytic vasculitis with deposition of fibrin and mucin (Fig. 2A).

Two years later, the patient reported months of intermittent left lower quadrant/periumbilical abdominal pain, and looser and frequent bowel movements. Skin lesions were stable. A 2-mm discrete, round sub-conjunctival white lesion with surrounding telangiectasiae suggested ophthalmic K–D.

Magnetic resonance enterography (MRE) revealed mild wall thickening of the terminal ileum (TI). Laparoscopic evaluation was advised by an expert on K–D. This demonstrated small white lesions scattered throughout the small and large bowel (Fig. 1D).

Systemic K-D was diagnosed and eculizumab was initiated two days post-operatively. Abdominal discomfort resolved after a few doses of eculizumab. Follow-up MRE showed improvement of his prior TI thickening and evidence of new mild inflammation of the descending colon. Two months later, subcutaneous treprostinil was initiated. His ocular lesion and skin lesions were stable. Repeat MRE while on dual therapy exhibited resolution of the colonic findings but mild active inflammation in a short segment of the TI.

Due to pain and irritation at the site of his treprostinil infusion, he transitioned to enteral treprostinil without any gastrointestinal side effects. Follow-up MRE obtained eight months from the prior study revealed ongoing mild ileal inflammaiton (as above) and a recurrence of hyperenhancement and inflammatory changes of the descending colon. This study notably followed some periods of missed eculizumab infusions due to challenges with insurance. Gastrointestinal symptoms recurred in this setting, though resolved when he resumed eculizumab consistently.

## CASE 2

A 16-year-old female presented with a two-year history of skin lesions. When evaluated, she had more than 50 lesions, each comprised of a central, porcelain atrophic scar surrounded by a rim of telangiectasia. The lesions spared the face, palms, and soles.

She reported generalized, progressively more severe and frequent, sharp abdominal pain with onset roughly concurrent with the skin lesions. Multiple emergency department evaluations resulted in an impression of functional abdominal pain.

Skin biopsy was highly suggestive of K-D, as revealed by attenuated epidermis, subjacent vascular drop-out, and fibrosis. Residual vessels within the fibrotic zone exhibited basement membrane zone thickening and were largely devoid of viable endothelium (Fig. 2B). The adjacent dermis demonstrated prominent mucin, lymphocytic vasculitis, and superficial vascular ectasia. There was striking upregulation of type I interferon signaling as revealed by extensive myxovirus protein resistance protein (MXA) staining (Fig. 2E). There were also prominent vascular deposits of C5b-9 (Fig. 2F).

**Fig. 3:**
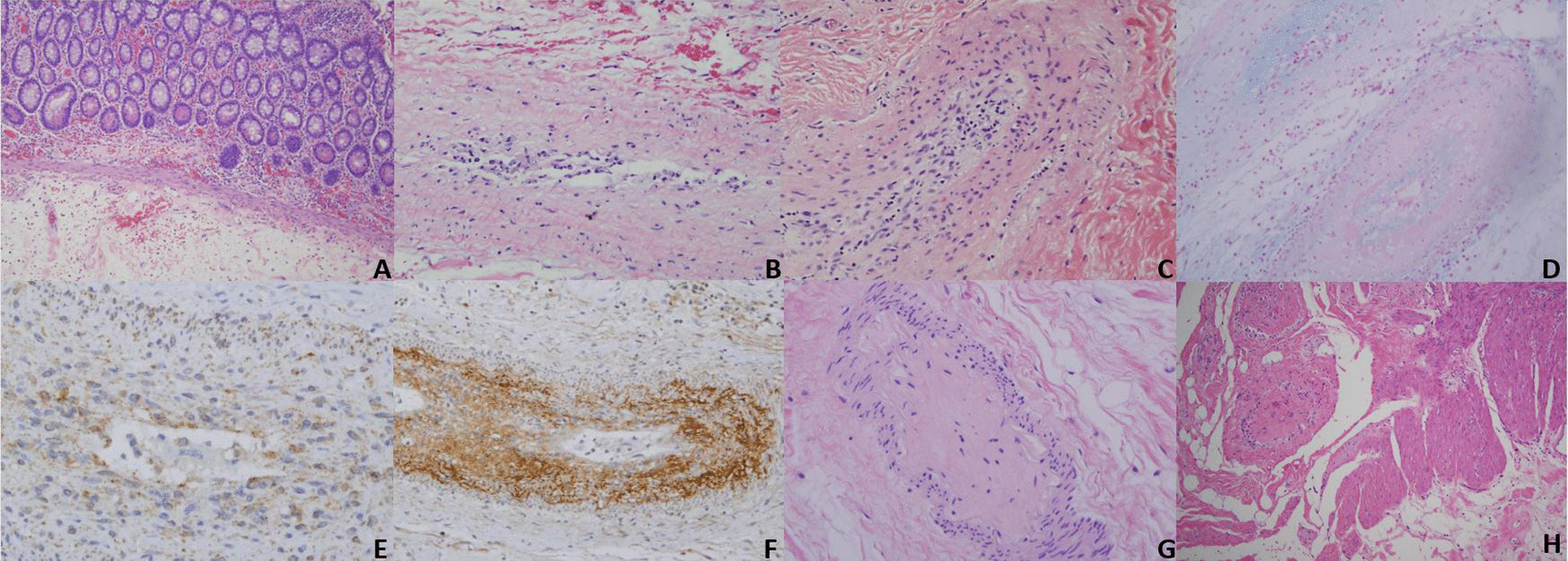
Chronologic pathology of gastrointestinal K-D as occurs in small- and medium-sized vessels within the subserosal fat: **A** Unlike the skin, no obvious small-vessel angiopathy of the mucosa occurs (H&E, ×10) **B** The incipient phase of the large-vessel arteriopathy is an accumulation of histiocytes and scavenger macrophages in the lumen and intima, along with intimal mucin deposition (H&E, ×10). **C** In the next phase of the progressive obliterative arteriopathy, the intima is expanded by inflammatory cells and mucin. There is variable fibrin deposition with some vessels showing a frank obliterative fibrin thrombus (H&E, ×10). **D** Reduction and progressive expansion of the intima occurs with a likely type I interferon effect as illustrated by the alcian blue stain (H&E, ×10). **E** MXA mirrors C5b-9 deposition (MXA, ×40) **F**. C5b-9 deposition (C5b-9, ×10). **G** Ultimately, the lumen is obliterated by loose matrix and a thrombus composed of collagen, mucin, and macrophages. This end-stage acellular collagenous plug obliterating the vascular lumen is highly specific to K-D (H&E, ×10). **H** Within the subserosa, an increase in collagen and mucin defines the key extravascular fibrosing and mucinous reaction of K-D (H&E, ×4)

While endoscopic evaluation of the gastrointestinal tract was normal, laparoscopy revealed multiple pale ulcerations on the serosal surface of the small bowel, most prominently at the TI. Full-thickness jejunal biopsy demonstrated lymphocytic and thrombogenic small vessel vasculitis while a striking mucinous obliterative intimal arteriopathy was observed in small- and medium-sized vessels (Fig. 3). A nonspecific chronic inflammatory infiltrate extended to the muscularis propria.

Given the escalating severity of abdominal pain, eculizumab was initiated. Significant advocacy was required to obtain this medication. After infusions were initiated, the patient reported almost immediate relief and sustained resolution of abdominal pain. She has not initiated treprostinil. Her skin lesions have remained stable.

## CASE 3

A 57-year-old female presented for evaluation of a three-month history of pruritic, progressively more numerous red bumps. On examination, there were multiple erythematous papules with mild scaling and central scarring scattered on the trunk and extremities, with predilection for the lower legs. Punch biopsy showed an attenuated epidermis with subjacent hyalinizing fibrosis and profound vascular drop-out. Within the hypovascular fibrotic zone, a few mummified-appearing capillaries and venules devoid of viable endothelium and exhibiting intravascular thrombosis were observed (Fig. 2C). Peripheral to this area of fibrosis was vascular ectasia and a brisk lymphocytic vascular reaction.

She had recently experienced new abdominal bloating but otherwise denied gastrointestinal symptoms. Endoscopy and sigmoidoscopy were normal.

Seven weeks later, she had a three-fold increase in skin lesions to over 100 in number. Diagnostic laparoscopy was strongly recommended. She began having severe abdominal pain several weeks before her surgery date.

An abdominal computed tomography revealed ascites and multiple foci of free air necessitating emergent laparotomy, where multiple ischemic lesions were visualized on the small bowel (Fig. 1C) along with perforation at the distal ileum. A partial small bowel resection and repair were performed. Pathology of the resected bowel revealed occlusive vasculopathy, ulceration, and necrosis.

Eculizumab was obtained from the manufacturer (Alexion Pharmaceuticals) and approved by the hospital for emergency use. During this time, the patient improved and was discharged.

Three days later, she returned with recurrent pain. A laparotomy was performed, revealing two new jejunal perforations distant from the previously resected region.

The following day, eculizumab was initiated, with a second infusion one week later. New cutaneous lesions continued to emerge. Her condition deteriorated, complicated by spiking fevers, respiratory failure, and abdominal free air suggestive of new perforations. She transitioned to comfort care 22 days into the admission and passed away the same day. The total time from cutaneous lesion onset to death was approximately eight months.

## Pathology of gastrointestinal K–D

Grossly, lesions in the gastrointestinal tract are similar to those on the skin: discrete erythematous macules that evolve into atrophic porcelain-white papules and plaques (Fig. 1B). The gross appearance of the skin and the gastrointestinal tract are similar, but the vascular pathology differs.

The thrombotic microvascular changes in the skin at the capillary venular level are not encountered in the lamina propria of the gastrointestinal tract (Fig. 3A). The disease in the gut is characterized by a purely arteriopathic and extravascular sclerosing process (Fig. 1H) confined to the subserosal fat. The small- and medium-sized blood vessels within the subserosal fat exhibit an expansile fibrosing neointimal arteriopathy eventuating in acellular collagen plugs.

The vascular changes include distinctive intimal proliferative arterial lesions. In the initial phase of the arteriopathy, there is influx of CD163+ CD11C+ monocytes into the intima, including foam cells and cells exhibiting a myofibroblastic phenotype. This influx is associated with the obliterative intimal arteriopathy and defines a critical event in lesional evolution. The infiltrating mononuclear cells demonstrate a myeloid dendritic cell phenotype revealed by the extent of CD4, CD14, and CD11C immunoreactivity. The cells express actin but not the smooth muscle markers desmin, calponin, and caldesmin. The arterial lesions resemble the obliterative intimal vasculopathy of lupus arteriopathy, coronary artery stent restenosis, and accelerated transplant rejection.

Temporal heterogeneity of gastrointestinal K-D is characteristic. An intestinal specimen can show these evolutionary distinct lesions. The incipient arterial lesion is one of asymmetrical expansion of the intima by mucin and collagen with a few mononuclear cells (Fig. 3B). Temporally, progressive collagen deposition and the inflammatory cell component becomes attenuated (Fig. 3C). The final phase of the proliferative arteriopathy is an acellular fibrous plug distinctive to K–D (Fig. 3G).

The larger vessel vasculopathy encountered in the subserosal blood vessels resembles the arterial changes that occur in dermatomyositis. In the latter, the distinctive serosal lesions of K–D are not seen (except when the two diseases coexist). MXA protein deposition provides evidence of enhanced interferon alpha expression in the pathogenesis of the obliterative arteriopathy of K–D (Fig. 3E). Although monokines and cytokines are critical to the pathology of both dermatomyositis and K–D, the microvascular and arteriopathic changes are often pauci-inflammatory in nature.

We feel that the neointimal proliferation intrinsic to the arterial lesion of K–D is an immunologically-driven process that reflects a proinflammatory milieu conducive to the influx of monocytes into the intima. The monocytes then undergo a transdifferentiation into matrix-producing myofibroblast-like cells. This could be pathogenetically implicated in the extravascular sclerosing pattern that resembles scleroderma.

Endothelial cell progenitor deficiency is likely a key contributor to the neointimal growth. When injured intima is devoid of endothelium, a lack of endothelial cell progenitor reconstitution results in a microenvironment conducive to monocyte recruitment and subsequent myofibroblastic differentiation.

The obliterative proliferative neointimal arteriopathy and the sclerodermic fibrosing serositis occur independently of complement inhibition (Fig. 3F) as we have seen both occur in patients exhibiting complete blockade of complement on eculizumab. Given the critical role of monocyte influx into the intima, enhancement of the type I interferon pathway is probably a key element in pathogenesis (Fig. 3D). In this regard, the operational profibrogenic pathways are potentially dependent on interferon-alpha but independent of complement.

## Treatment

Until recently, virtually all published cases of systemic K-D reported catastrophic and fatal outcomes. Deaths from gastrointestinal K-D resulted from recurrent bowel perforation, with hemorrhage, hypotension, and sepsis. In our review, 59% (19/32) of those with gastrointestinal perforations died of sepsis or severe peritonitis [2, 4–6, 10, 15, 17, 19, 20, 24, 27, 30, 31, 55].

While single case reports of remission with other therapies exist [4, 13, 54], prolonged remission of symptoms from gastrointestinal K–D has been achieved with combined therapy with eculizumab [61] and treprostinil [62].

Individuals diagnosed with K–D only after presenting with bowel perforation require urgent intervention. In addition to the complications attendant on the perforation, they are at high risk of additional perforation(s) due to the multiplicity of bowel lesions. Eculizumab should be infused as soon as possible. The onset of action is immediate, but the outcome may be poor because of sepsis and vascular instability that can often result in the setting of bowel perforation.

Those with laparoscopic identification of lesions typical of K–D but without an acute abdomen must be considered at high risk of perforation and the initiation of eculizumab should not be delayed. Those patients who have not previously received meningococcal vaccination will require prophylactic antibiotics.

Any individual with K–D experiencing unexplained abdominal pain warrants immediate performance of laparoscopy. This cannot be overemphasized. Based on only small numbers to date, our experience suggests no other procedure has comparable sensitivity or specificity. No other disorder produces similarly-appearing serosal lesions (except possibly peritoneal mesothelioma, unpublished comments Andrew Blakely, MD). Hesitancy to perform a surgical procedure for diagnosis puts the patient at high risk for diagnosis made only after perforation.

There has only been one report of fatality from gastrointestinal K–D when eculizumab and treprostinil were initiated before bowel perforation (verbal communication Joseph Beck, MD).

Monotherapy with eculizumab, remarkably effective as a gastrointestinal crisis intervention, has not proven to provide long-term symptomatic relief. Within months of initiation of treatment, those so treated have noted recurrent abdominal pain. Perforation while on long-term eculizumab monotherapy has been seen [37].

Treprostinil, a prostacyclin analogue, was first employed in systemic K–D after a patient with K–D-like lesions in the setting of scleroderma-lupus overlap noted regression of her cutaneous lesions after subcutaneous treprostinil was initiated for pulmonary hypertension [3]. In addition to vasodilating and antithrombotic properties, treprostinil has been reported to increase the number of endothelial progenitor cells in children with idiopathic pulmonary hypertension [63].

Addition of treprostinil by subcutaneous or oral route has resulted in prolonged remission of gastrointestinal symptoms when added to eculizumab [58]. Treprostinil must be slowly titrated up to therapeutic dose and thus is no substitute for eculizumab in the acute setting of gastrointestinal K–D. There is no report of successful outcome with treprostinil monotherapy.

Although subcutaneous (or intravenous) treprostinil has more consistent absorption than when administered orally, subcutaneous administration is often poorly tolerated with intense infusion site pain. In the setting of recurrent gastrointestinal K–D, we favor initiating subcutaneous treprostinil and considering subsequent conversion to oral therapy for patient convenience and tolerance.

At present, there is no serologic marker of K–D activity and no imaging study that has been shown to be highly specific for disease activity. Drug response has been assessed clinically, primarily by resolution of abdominal pain. However, individuals with K–D who have experienced bowel perforation and developed adhesions may have recurring pain even in the absence of active disease. Repeating laparoscopy to document involution or resolution of serosal lesions may be the best objective means to demonstrate drug efficacy, but this has been infrequently performed. The authors believe laparoscopic evaluation is the most powerful means of both diagnosing gastrointestinal K–D and of assessing response to therapy.

The combination of eculizumab and treprostinil has failed to prevent the emergence or progression of neurologic or pleural-pericardial disease in some individuals.

In contrast to use of eculizumab in atypical hemolytic uremic syndrome (aHUS), therapy is needed indefinitely for K–D. Insurance coverage, essential for access, may be difficult to secure and maintain because of drug expense, the “off-label” nature of this use, and the lack of double-blind controlled studies demonstrating efficacy. In addition, many case-reviewers may be unfamiliar with this ultra-rare disease. For those uninsured or under-insured, for individuals in third-world countries, or for those with national health insurance, the expense of therapy, although potentially lifesaving, may be an insurmountable barrier.

The case for drug approval rests on a few key elements. First, untreated systemic K–D is a rapidly fatal disease and reference can be made to the other name of this disorder: malignant atrophic papulosis. Second, if treatment for this disorder were restricted to FDA-approved therapy, there would be no treatment. Third, the ultra-rare incidence of this disorder and the fatal nature of untreated disease mean that double-blind controlled studies are neither feasible nor ethical. At best, we can compare outcome on current therapy with the dreadful outcomes reported in the literature prior to current therapy. Fourth, prolonged survival of a cohort of patients on this two-drug regimen has now been reported.

The therapies discussed will undoubtedly undergo evolution as disease mechanisms are better understood. There are now drugs that directly target the interferon pathway and others with anti-interferon activity. Finally, given the identical morphology with transplant rejection and accelerated atherosclerosis, drugs that reduce neointimal proliferation such as rapamycin might be added to the regimen; but there is no report of the use of these drugs yet.

## Conclusion

Unrecognized and untreated gastrointestinal K–D will result in perforation and death. Yet, there is almost always opportunity for diagnosis and intervention before such catastrophic outcomes.

First and foremost, there must be greater awareness of K–D among dermatologists and gastroenterologists. The lesions have such a characteristic appearance that familiarity with the entity should result in its prompt recognition. Dermatologists must educate individuals diagnosed with K–D of the risk of progression to systemic disease and inform them that the gastrointestinal tract is the most common system involved, encouraging follow-up should gastrointestinal symptoms arise. In individuals with unexplained gastrointestinal symptoms, the gastroenterologist should always look for cutaneous lesions typical of K–D. The patient may be unaware of their existence and the lesions may be few in number.

Based on review of the literature, gastrointestinal symptoms preceded perforation. Nevertheless, providers should remain vigilant for perforation as symptom minimization is a potential consideration. Of note, we are aware of one unpublished case in which a patient with long-standing skin lesions suffered perforation without any reported preceding gastrointestinal symptom. Because the pathology of the disease centers on the bowel serosa, laparoscopy is the best means to early discovery of bowel involvement. In the individual with unexplained gastrointestinal symptoms in the setting of K–D, laparoscopy should not be delayed. If lesions consistent with K–D are identified on the bowel serosa, treatment with eculizumab should be initiated promptly. Eculizumab has immediate onset of action but its benefit may not suffice to prolong the lives of those who have already sustained perforation.

Monotherapy with eculizumab does not result in long-term remission. Addition of treprostinil is necessary to maintain remission from gastrointestinal K–D. However, there are reports of progressive neurologic, pleural, and pericardial K–D despite ongoing therapy with eculizumab and treprostinil. Further therapies to address and reverse the neointimal proliferation associated with K–D will undoubtedly emerge. Systemic K–D, malignant atrophic papulosis, is increasingly a treatable disease, particularly when promptly recognized and diagnosed.

## Supplementary Information

**Additional file 1.** **Appendix 1.** EBSCOhost was used to search Business Source Elite; CINAHL; EBSCOhost eBook Collection; ERIC; GreenFILE, Health Course; Nursing/Academic Edition; Library, Information Science and Technology Abstracts; MEDLINE; Newspaper Source; Professional Development Collection; APA PsychArticles; APA PsychInfo; Regional Business News; and The Serials Directory. Only Academic Search Elite, Health Source: Nursing/Academic Edition, and APA PsychInfo yielded articles based on search terms.

## Data Availability

Not applicable.

## Abbreviations

K–D: Kohlmeier–Degos
TI: Terminal ileum
MXA: Myxovirus protein resistance protein
aHUS: Atypical hemolytic uremic syndrome
