# Supplementary material for: Gastrointestinal Kohlmeier–Degos disease: a narrative review
Source: Orphanet J Rare Dis. 2022 Apr 20;17:172. doi: 10.1186/s13023-022-02322-9 (PMC9022239; doi:10.1186/s13023-022-02322-9)
Supplement: Supplementary file 1 — Additional file 1. Appendix 1. EBSCOhost was used to search Business Source Elite; CINAHL; EBSCOhost eBook Collection; ERIC; GreenFILE, Health Course; Nursing/Academic Edition; Library, Information Science and Technology Abstracts; MEDLINE; Newspaper Source; Professional Development Collection; APA PsychArticles; APA PsychInfo; Regional Business News; and The Serials Directory. Only Academic Search Elite, Health Source: Nursing/Academic Edition, and APA PsychInfo yielded articles based on search terms. [file 13023_2022_2322_MOESM1_ESM.docx]

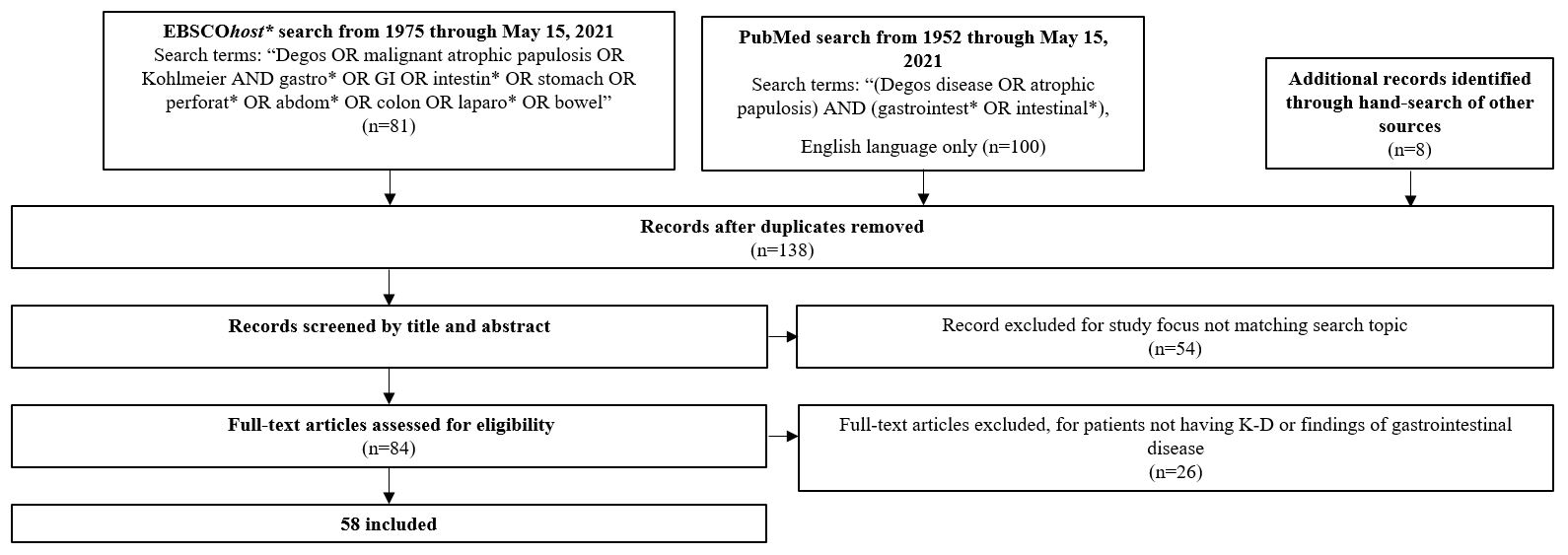


**Appendix 1**. EBSCOhost was used to search Business Source Elite; CINAHL; EBSCOhost eBook Collection; ERIC; GreenFILE, Health Course; Nursing/Academic Edition; Library, Information Science and Technology Abstracts; MEDLINE; Newspaper Source; Professional Development Collection; APA PsychArticles; APA PsychInfo; Regional Business News; and The Serials Directory. Only Academic Search Elite, Health Source: Nursing/Academic Edition, and APA PsychInfo yielded articles based on search terms.
